# Betaine‐assisted recombinase polymerase assay for rapid hepatitis B virus detection

**DOI:** 10.1002/bab.1940

**Published:** 2020-06-30

**Authors:** Ting‐ting Yi, Han‐yun Zhang, Hua Liang, Guo‐zhong Gong, Yan Cai

**Affiliations:** ^1^ Department of Clinical Laboratory Affiliated Hospital of North Sichuan Medical College Nanchong Sichuan People's Republic of China; ^2^ Department of Laboratory Medicine North Sichuan Medical College Nanchong Sichuan People's Republic of China; ^3^ Zhongshan School of Medicine Sun Yat‐sen University Guangzhou People's Republic of China; ^4^ Sichuan CellMed Clinical Laboratory Co., Ltd Nanchong Sichuan People's Republic of China; ^5^ Department of Laboratory Medicine Suining First People's Hospital Suining People's Republic of China; ^6^ Prenatal diagnosis center Affiliated Hospital of North Sichuan Medical College Nanchong Sichuan People's Republic of China

**Keywords:** betaine, hepatitis B virus, lateral flow assay, recombinase polymerase amplification

## Abstract

Hepatitis B virus (HBV) is a worldwide epidemic pathogen that causes hepatitis B. On‐site screening the HBV infection is of critical importance for preventing and diagnosing HBV infection. In this paper, a simple, visual, and rapid method for on‐site detection of HBV‐DNA has been developed. This method is based on betaine‐assisted recombinase polymerase assay and followed with naked‐eye detection via lateral flow assay (BRPA‐LF). Result show that nonspecific amplification is prone to occur in recombinase polymerase amplification (RPA) if the assay was performed with serum sample without purification. This problem has been addressed by adding 0.8 M of betaine to the RPA reactions. It was demonstrated that BRPA‐LF can detect 1,000 copies of HBV‐DNA in 50 μL mixture, and achieved 90% sensitivity and 100% specificity for serum sample detection. These results demonstrated that BRPA‐LF can resist serum interference and has great potential for on‐site screening of HBV infection.

## Graphical abstract

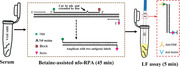

**Abbreviations**
BARPA: Betaine‐assisted recombinase polymerase assay
BRPA‐LF: betaine‐assisted recombinase polymerase‐lateral flow
DNA: desoxyribonucleic acid
HBV: hepatitis B virus
NTC: no template control
PAGE: polyacrylamide gel electrophoresis
PCR: polymerase chain reaction
pHBVS: S‐protein gene from HBV
POCT: point‐of‐care testing
RPA: recombinase polymerase amplification

## 1 Introduction

Hepatitis B virus (HBV) is a blood borne pathogen that causes hepatitis, liver cirrhosis, and hepatocellular carcinoma [1]. Worldwide, an estimated 2 billion people have been infected with the HBV, and about 257 million people suffer from chronic HBV infection [2]. Early diagnosis and immediate treatment of HBV infection remains a public health priority.

Although the diagnosis of HBV infection is usually made by serologic methods, HBV‐DNA detection in serum is important for diagnosing early acute HBV infection, distinguishing active from inactive HBV infection and monitoring a patient's response to anti‐HBV therapy [3]. Further, the presence of transplacentally acquired maternal antibodies make serologic testing unreliable in young infants [4]. Consequently, the diagnosis of HBV infection in young infants currently relies on HBV‐DNA detection.

Polymerase chain reaction (PCR) is the most frequently used testing method for HBV‐DNA detection [3]. PCR is reasonably useful, due to its high sensitivity and specificity. However, the requirement of skilled personnel and expensive equipment make PCR only be available in laboratory setting [5]. Hence, in resource‐limited area, more convenient and cost‐effective methods for HBV‐DNA detection are highly desired.

Recombinase polymerase amplification (RPA) is one of the most popular isothermal amplification methods in modern biological and medical sciences [6]. Since its invention in 2006, RPA have been widely used for nucleic acid testing and regarded as an excellent candidate to replace PCR. RPA employs two opposing primers and three proteins (RecA, SSB, and SauDNA polymerase), which are used to replace thermal cycling process of PCR, to exponentially amplify a template DNA [7]. RPA is robust, convenient, and can be carried out under a wide temperature range. These traits make RPA to be a useful point‐of‐care testing (POCT) tool for molecular diagnosis. So, the application of RPA for on‐site screening of HBV infection with serum sample may be a feasible plan.

In this paper, we report a visual POCT method for HBV‐DNA detection, which based on betaine‐assisted recombinase polymerase assay and followed with naked‐eye detection via lateral flow assay (BRPA‐LF). BRPA‐LF is rapid, robust, visual, and useful for on‐site screening of HBV infection.

## 2 Methods

### 2.1 Materials and reagents

All the oligonucleotide listed in Table 1 were designed by Primer Premier 6 software and synthesized by Sangon Biotech (Shanghai, China). Lyophilized TwistAmp^®^ nfo‐RPA kits were purchased from TwistDx Limited (Cambridge, UK). Lateral flow strips were purchased from Milenia Biotech (Gießen, Germany). DHelix real‐time incubator was purchased from DHelix (Guangzhou, China). Betaine was purchased from Aladdin Company (Shanghai, China). The 50 and 20 bp DNA markers and chemicals used to prepare electrophoresis were purchased from Takara Bio (Beijing, China).

**TABLE 1:** Primer and probe for TwistAmp‐nfo‐RPA

Name	Primer sequence (5'–3')	Primer length	Product length
**P1**	P1F: AACCTCCAATCACTCACCAACCTCT	25 bp	134
	P1B: Bio‐GATAGTCCAGAAGAACCAACAAGAAGA	27 bp	
	Probe1: Fam‐GATGTGTCTGCGGCGTTTTATCATATTCCTC(THF)TCATCCTGCT‐P	42 bp	
**P2**	P2F: CCTCCAATCACTCACCAACCTCTTGTCCTC	30 bp	145
	P2B: Bio‐GGCAACATACCTTGATAGTCCAGAAGAACCA	31 bp	
	Probe2: Fam‐ATAGCAGCAGGATGAAGAGGAATATGATAAAACG(THF)CGCAGACACA‐P	45 bp	
**P3**	P3F: TTATCATATTCCTCTTCATCCTGCTGCTATGC	32 bp	115
	P3B: Bio‐GTGCTGGTAGTTGATGTTCCTGGAAGTAGA	30 bp	
	Probe3: Fam‐TCTTCTTGTTGGTTCTTCTGGACTATCAAGGTA(THF)GTTGCCCGTT‐P	44 bp	
**P4**	P4F: AAACTGCACTTGTATTCCCATCCCATCATC	30 bp	163
	P4B: Bio‐CCACATCATCCATATAACTGAAAGCCAGACA	31 bp	
	Probe4: Fam‐GATTCCTATGGGAGTGGGCCTCAGTCCGTTTCTCC(THF)GGCTCAGT‐P	44 bp	

### 2.2 Template DNA for nfo‐RPA

Both plasmid DNA with HBV fragment and clinical serum samples with HBV positive were used as template for RPA reaction. The plasmid DNA contained a 680 bp fragment of S‐protein gene from HBV (pHBVS), which was inserted into a pUC57 plasmid (2,710 bp). A total of 40 donor serum samples (20 HBV positive and 20 HBV negative) were also used for clinical practicability evaluation. The DNA concentration of plasmid DNA was determined by Multiskan Spectrophotometer (ThermoScientific, Waltham, MA, USA). The copy number of pHBVS was calculated using the following equation: DNA copy number = (ng × 6.02 × 10^23^ × 10^−9^)/(fragment length [bp] × 660). The HBV viral load of clinical serum samples were detected by commercial real‐time qPCR HBV‐DNA detection Kit (Yaneng Bio, Shenzhen, China)

### 2.3 nfo‐RPA and betaine‐assisted nfo‐RPA

The nfo‐RPA reaction was performed in a 50 μL final volume by using a TwistAmp‐nfo‐lyophilized kit. According to the manufacturer's instructions, all reagents except the template and Mg acetate were prepared in a master mixture and distributed into 0.2 mL reaction tubes, then 2.5 μL of Mg acetate and 2 μL of the template were added to the master mixture. The reaction tubes were vigorously mixed and immediately incubated in a DHelix real‐time incubator at 38 °C for 45 Min. To address the nonspecific amplification of nfo‐RPA in serum sample, we added betaine to the nfo‐RPA reactions (BRPA). The final concentration of betaine in nfo‐RPA reaction mixture was 0.8 M.

### 2.4 Product analyzed by electrophoresis

For electrophoretic analysis, nfo‐RPA or B‐ nfo‐RPA was performed an identical manner, except that the nfo‐probe was omitted. A total of 5 μL of products were electrophoresed by 12% polyacrylamide gel electrophoresis (PAGE). Then, the polyacrylamide gel was stained with ethidium bromide (Sangon Biotech) and visualized with the ChemiDoc^TM^ XRS Imaging System (Bio‐Rad, Hercules/California, USA).

### 2.5 Product analyzed by lateral flow assay

nfo‐RPA or BRPA was performed at 38 °C for 45 Min, then the reaction tubes were immediately incubated at 95 °C for 10 Min to terminate amplification. Prior to lateral flow assay, 2 μL of each stopped reaction was diluted by 98 μL lateral flow buffer. The lateral flow strips were placed in the mixture, then the results were read between 5 and 10 Min. Positive results were formed a red band on the test line and control line respectively, whereas negative results displayed only the control line.

## 3 Results

### 3.1 Primer pair screening

**FIG. 1:**
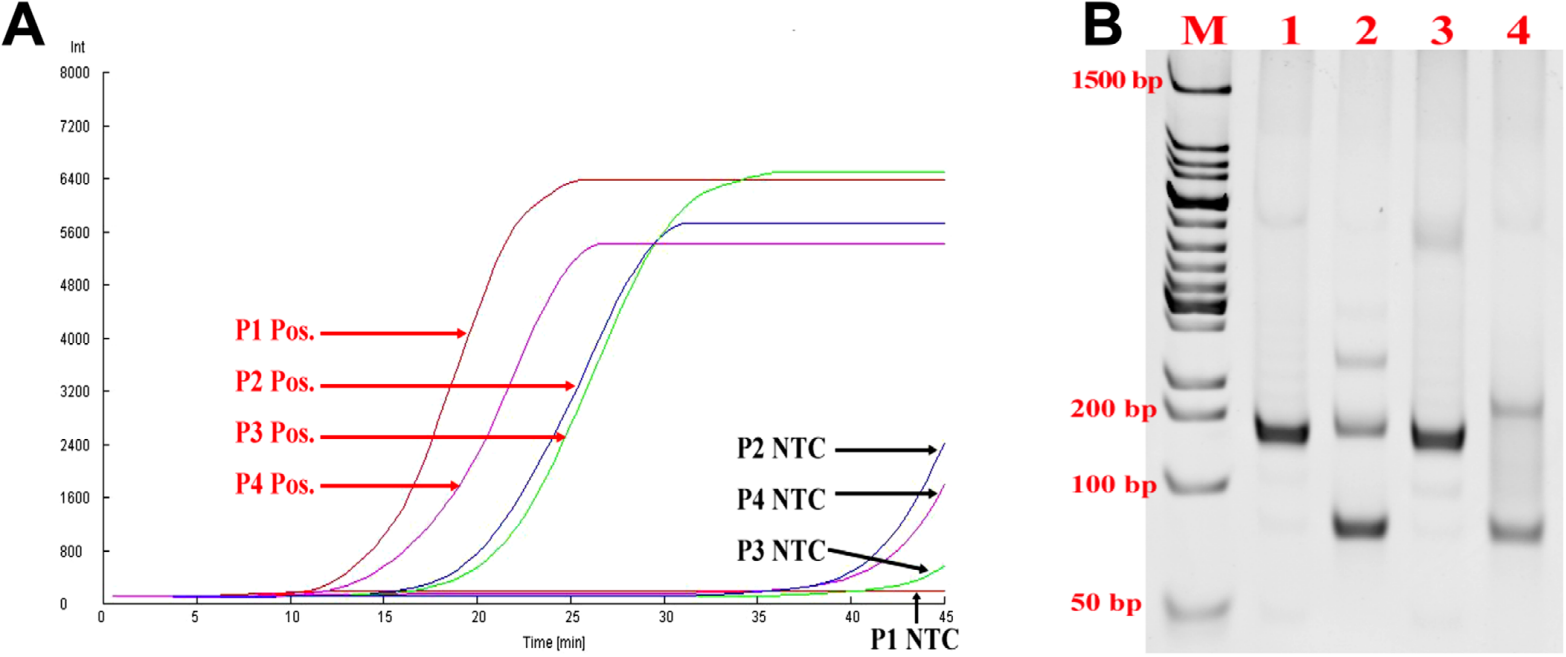
Primer pair screening. (A) Real‐time nfo‐RPA for primer screening. P1 showed the best performance in amplification speed and NTC. Pos. refers to the reaction contained 2 × 10^8^ copies of pHBVS. NTC refers to no template control. (B) The specificity of amplificon was analyzed by PAGE. Five microliters of the positive product was analyzed by 12% PAGE, and only the P1 amplificon showed an exclusive band.

First of all, to screen the high‐efficiency primer for HBV‐DNA detection, four pairs of nfo‐RPA primers and corresponding nfo‐probe were designed (Table 1). As shown in Fig. 1A, the nfo‐RPA with P1 successfully amplified 10^8^ copies of plasmid DNA with HBV fragment (pHBVS) and showed the best efficiency and no template control (NTC). To confirm the specificity of products, 5 μL of the nfo‐RPA product was analyzed by 12% PAGE. The results showed that an exclusive band at the expected size range was primed by P1 primer pairs, and many nonspecific bands existed in the products primed by P2–P4 (Fig. 1B).

### 3.2 RPA for serum sample detection

**FIG. 2:**
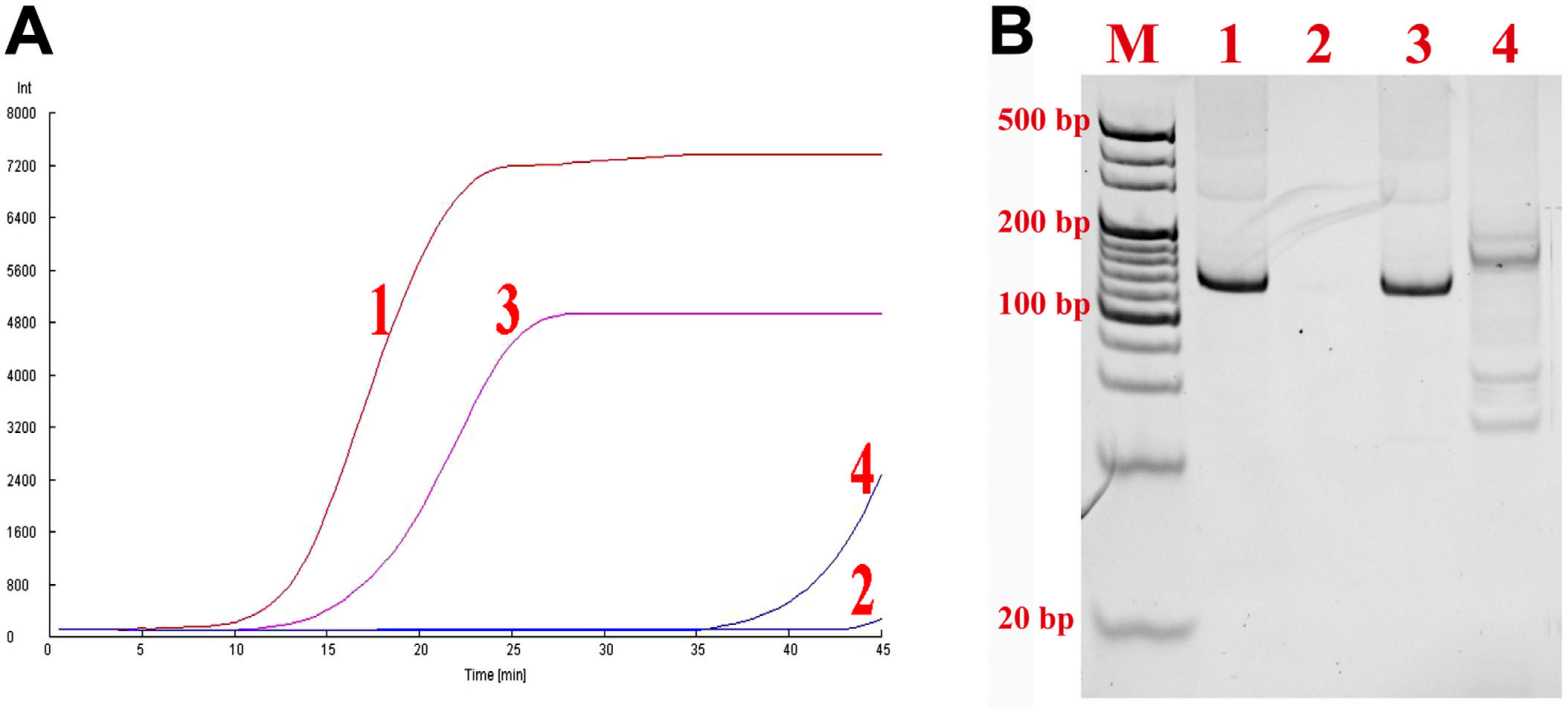
nfo‐RPA for serum sample detection. (A) Real‐time analysis of nfo‐RPA. Line 1, positive sample without serum; line 2, negative sample without serum; line 3, positive sample with serum; line 4, negative sample with serum. (B) The PAGE analysis with the reaction samples in panel A.

To confirm the clinical practicability, the nfo‐RPA was investigated with the pHBVS samples spiked by serum (2 μL of serum was added to 48 μL of reaction mixture). In the absence of serum, nfo‐RPA can detect the target DNA (2 × 10^8^ copies of pHBVS in 50 μL reaction) rapidly and no nonspecific amplification occurred in negative control. On the contrary, in the presence of serum, the threshold time of positive sample was delayed and nonspecific amplification occurred in negative control (Fig. 2A). Further, PAGE analysis confirmed the nonspecific amplification of nfo‐RPA in serum sample (Fig. 2B).

### 3.3 Betaine‐assisted nfo‐RPA with enhanced specificity for serum sample detection

**FIG. 3:**
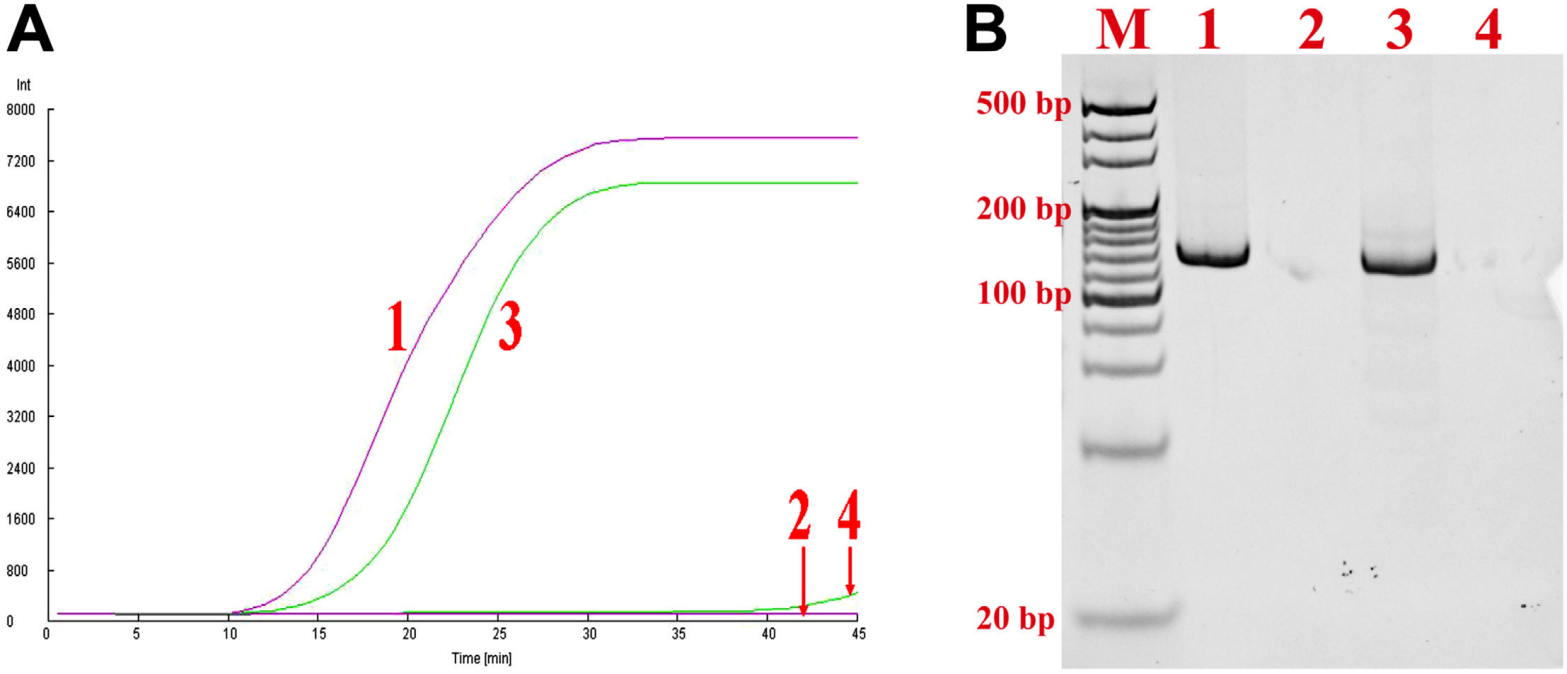
BRPA for serum sample detection. (A) Real‐time analysis of BRPA. Line 1, positive sample without serum; line 2, negative sample without serum; line 3, positive sample with serum; line 4, negative sample with serum. (B) The PAGE analysis with the reaction samples in panel A.

Our previous study demonstrated that the addition of betaine (0.8 M) to the basic‐RPA can reduced nonspecific amplification caused by background DNA [6]. So, in this study, the addition of betaine to the nfo‐RPA was used to address nonspecific amplification caused by serum (BRPA). As shown in Fig. 3A, the nonspecific amplification occurred in nfo‐RPA for serum sample detection was alleviated significantly by addition of 0.8 M betaine. PAGE analysis confirmed that nonspecific bands were virtually not observed under the same conditions (Fig. 3B).

### 3.4 Sensitivity and specificity of BRPA

**FIG. 4:**
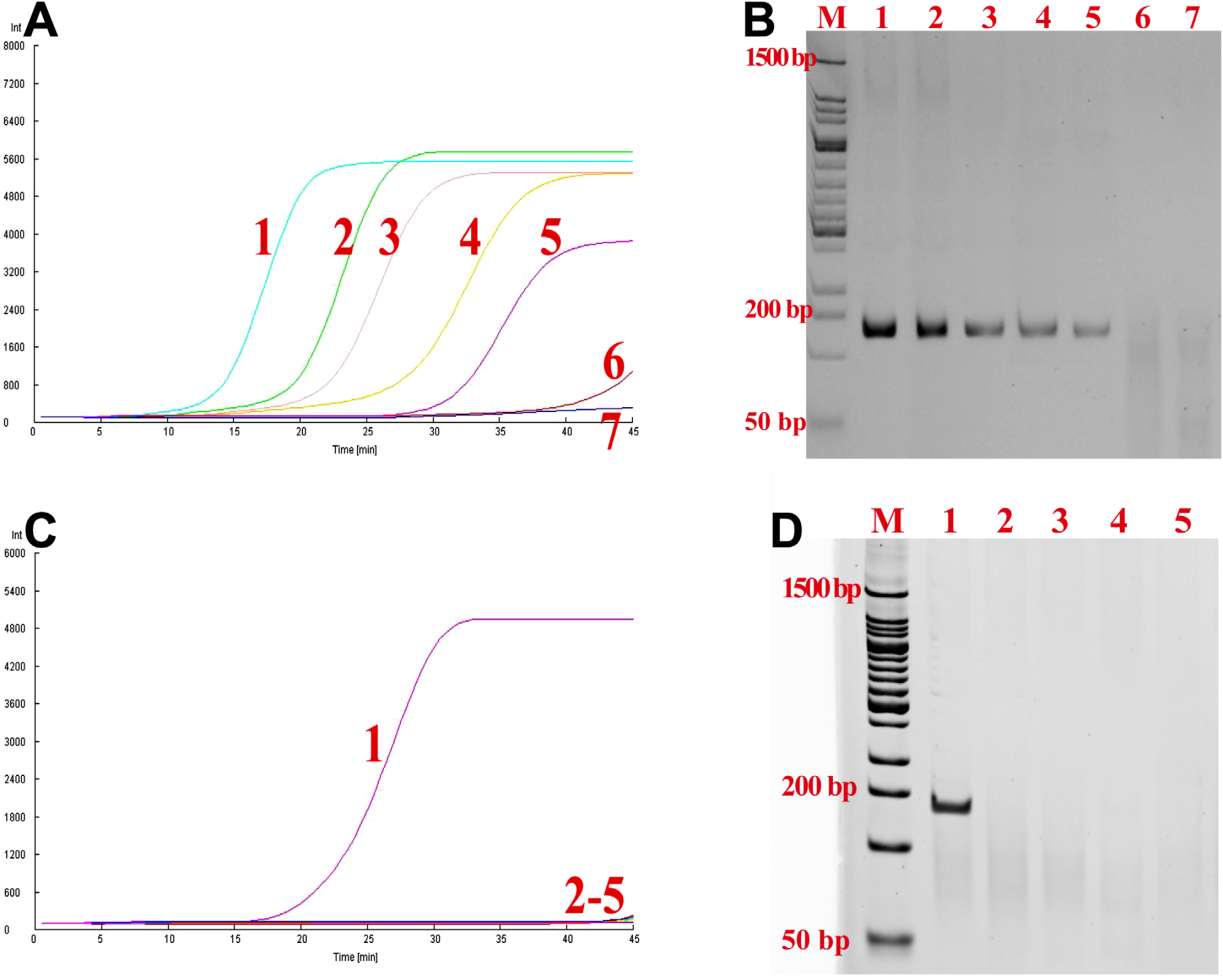
Sensitivity and specificity of BRPA. (A) The sensitivity of BRPA. Line 1 to line 6 referred to 10^6^ to 10^1^ copies of pHBVS in 50 μL reaction. Line 7 referred to negative control. (B) The PAGE analysis with the reaction samples in panel A. (C) The specificity of BRPA. Line 1, HBV positive sample; line 2, EBV positive sample; line 3, CMV positive sample; line 4, HSV positive sample; line 5, HCV positive sample. (D) The PAGE analysis with the reaction samples in panel C.

The analytical sensitivity of the BRPA was evaluated by testing serial dilutions of the pHBVS range from 10^1^ to 10^6^ copies per 50 μL reaction. As shown in Figs. 4A and 4B, the BRPA can detect 100 copies of target DNA in 50 μL reaction mixture. The specificity of the BRPA was investigated by testing serum samples with EBV, CMV, EBV, and HCV positive, respectively. As shown in Figs. 4C and 4D, no nonspecific amplification was occurred in samples with EBV, CMV, EBV, and HCV positive.

### 3.5 BRPA products analyzed by LF assay

**FIG. 5:**
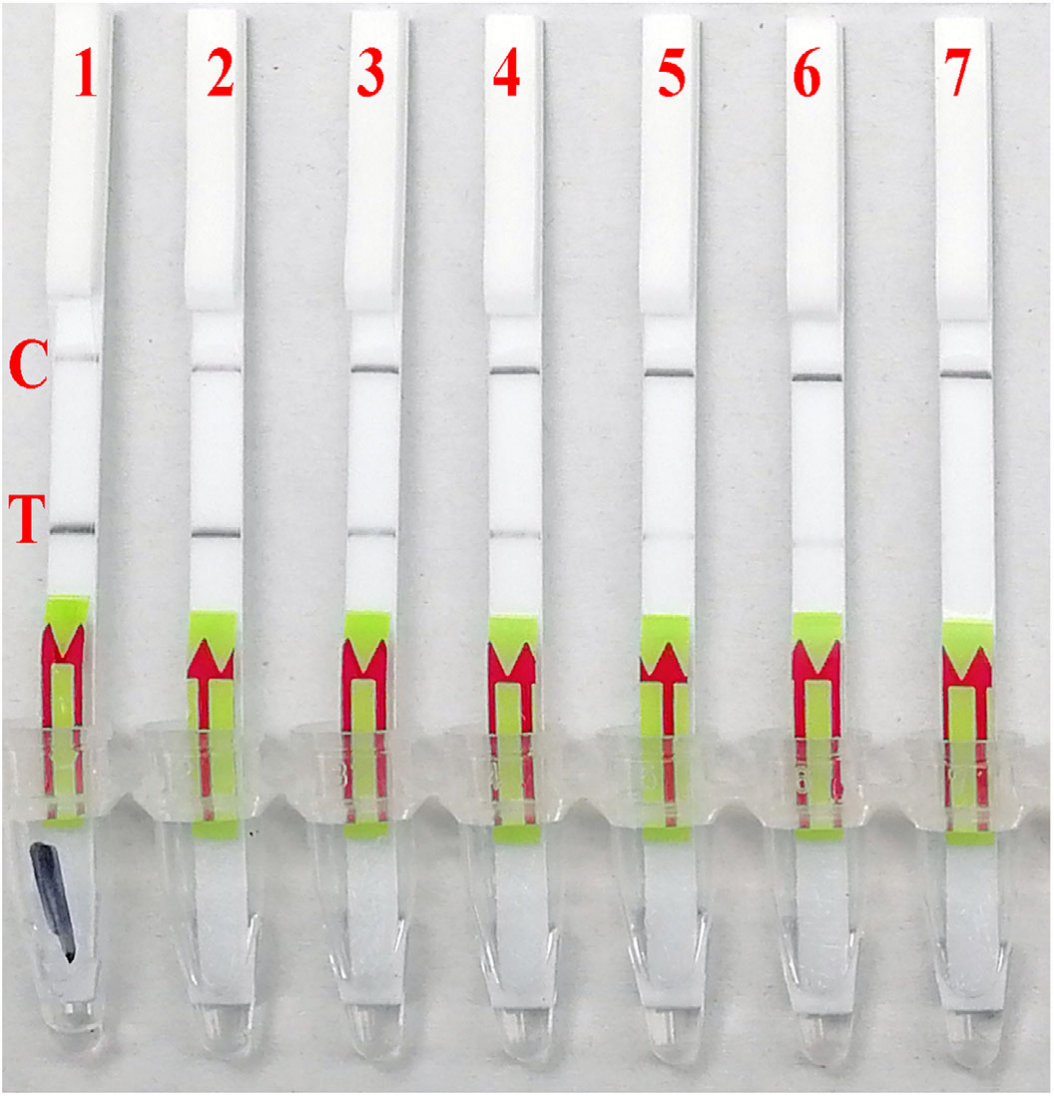
LF assay for BRPA product analysis. The BRPA‐LF can detect 10^3^ copies of pHBVS in 50 μL reaction. Strip 1, 10^6^ copies of pHBVS; strip 2, 10^5^ copies; strip 3, 10^4^ copies; strip 4, 10^3^ copies; strip 5, 10^2^ copies; strip 6, 10 copies; strip 7, negative control.

In order to facilitate the on‐site application of BRPA, the LF strips were used for naked‐eye observation (BRPA‐LF). The BRPA products can be efficiently detected by LF strip and the sensitivity of LF analysis was slightly dropped, which can detect 1,000 copies of pHBVS in 50 μL reaction mixture (Fig. 5).

To demonstrate the clinical practicability, we also performed BRPA‐LF on 40 serum samples. The HBV‐DNA load of these samples was firstly tested by qPCR, and 20 of them were tested as positive (HBV‐DNA > 500 IU/mL; Table 2). Testing results from BRPA‐LF showed that 18 of 20 positive and all negative samples were correctly identified by BRPA‐LF, indicating 95% consistency with the qPCR method and suggesting its potential as a simple, rapid, robust, and useful tool for POCT.

**TABLE 2:** BRPA‐LF for clinical serum sample detection

Sample	qPCR (IU/mL)^a^	BRPA‐LF		Sample	qPCR (IU/mL)	BRPA‐LF
P1	2.27+E5	**++**		N1	<500	**—**
P2	1.50+E8	**+++**		N2	<500	**—**
P3	1.42+E5	**++**		N3	<500	**—**
P4	1.70+E8	**+++**		N4	<500	**—**
P5	1.54+E7	**+++**		N5	<500	**—**
P6	5.25+E5	**+**		N6	<500	**—**
P7	2.36+E3	**+**		N7	<500	**—**
P8	1.77+E3	±^b^		N8	<500	**—**
P9	5.58+E4	**+**		N9	<500	**—**
P10	1.44+E3	**—**		N10	<500	**—**
P11	3.87+E6	**++**		N11	<500	**—**
P12	2.38+E6	**++**		N12	<500	**—**
P13	2.07+E3	**+**		N13	<500	**—**
P14	6.75+E4	**+**		N14	<500	**—**
P15	5.85+E7	**+++**		N15	<500	**—**
P16	4.56+E5	**++**		N16	<500	**—**
P17	3.58+E7	**+++**		N17	<500	**—**
P18	3.27+E7	**+++**		N18	<500	**—**
P19	4.32+E4	**+**		N19	<500	**—**
P20	2.00+E6	**++**		N20	<500	**—**

a The qPCR results were detected by commercial real‐time qPCR HBV‐DNA detection Kit (Yaneng Bio). 1 IU/mL ≈ 5.6 copy/mL.

b The P8 showed weak test line on the strip and was defined as negative.

## 4 Discussions

The results of this study showed that nonspecific amplification was prone to occur in nfo‐RPA if the assay performed with serum sample. With the addition of inexpensive betaine, the nonspecific amplification of the nfo‐RPA significantly suppressed. This improved nfo‐RPA was named BRPA. To facilitate the on‐site application of BRPA, the LF assay was used for product analysis. The BRPA‐LF assay showed 95% consistency with the qPCR method for serum sample detection, thereby demonstrating its potential as a simple, rapid, and robust tool for on‐site detection of HBV‐DNA.

RPA is becoming one of the most popular isothermal amplification methods in nucleic acid testing due to its simplicity, rapidity, sensitivity, and operation at a low and constant temperature [7]. RPA has been developed various types, one of which is TwistAmp nfo‐RPA kit for real time or end point detection. The nfo‐RPA needs a forward primer, a 5′‐end biotin‐labeled reverse primer and a special probe. The nfo‐probe is labeled with FAM at 5′‐end and blocked with phosphate group at 3′‐end and substituted one of the internal bases with nucleotide analogue (THF). The amplicon of nfo‐RPA will include two labels and ready to be visually detected by lateral flow assay in a sandwich format by antibodies or streptavidin.

Despite RPA is a useful tool for molecular diagnosis, nonspecific amplification is a major limitation [6, 8‐10]. Zhang et al. [11] demonstrated that RecA‐ssDNA filament can tolerate a maximum mismatch of 18 bases in an 80‐mer DNA. Daher et al. [8] further reported that the mismatch discrimination by RPA is unsuccessful when the target DNA polymorphism is <36%. Boyle et al. [12] reported that RPA can tolerate up to nine mismatches across the primer and probe regions. Our previous study demonstrated that nonspecific amplification is prone to occur in RPA if the sample contains high background DNA [6]. The present study showed that nonspecific amplification is also prone to occur in nfo‐RPA if the assay performed with serum sample. Thus, serum sample without purification is not suitable for nfo‐RPA amplification.

Betaine is a known PCR additive. As a denaturant, betaine can decrease the melting temperature of dsDNA and enhance the specificity of isothermal amplification reactions [13]. In addition, betaine is also used as a molecular barrier to hinder the nonspecific hybridization between the primer and background DNA, thereby increasing the specificity of isothermal amplification [14]. In this study, we employed betaine to alleviate the nonspecific amplification of nfo‐RPA, and the results showed that the addition of 0.8 M betaine can significantly enhance the specificity of nfo‐RPA in serum sample detection. This betaine‐assisted nfo‐RPA can detect 100 copies of HBV‐DNA in 50 μL reaction in serum sample. Furthermore, LF assay was employed to detect the nfo‐RPA products by naked‐eye observation. The BRPA‐LF assay showed 95% accuracy for serum sample detection, indicating its capacity for resisting serum interference. In addition, the whole process of BRPA‐LF assay could be completed in one hour. These results suggested that BRPA‐LF is rapid, robust, visual, and useful for on‐site screening of HBV infection.

In conclusion, nonspecific amplification was prone to occur in nfo‐RPA‐FL assay for serum sample detection. However, the addition of economical betaine can suppress the nonspecific amplification significantly. The BRPA‐LF assay showed 95% consistency with the clinically approved qPCR method for serum sample detection, thereby demonstrating its potential for on‐site nucleic acid detection and offering an alternative strategy for initial screening of HBV infection. However, the testing result of BRPA‐LF still need to be identified by clinically‐approved method.

## 6 Conflict of Interest

The authors declare no conflict of interest.
